# Synthesis of novel and sustainable molybdenum disulfide (MoS_2_)-doped biomass-carbon adsorbent with high-performance sodium diclofenac removal

**DOI:** 10.1039/d6ra03342c

**Published:** 2026-07-21

**Authors:** Wenyuan Duan, Yanlin Li, Lashari Najeeb ur Rehman, Irineu A. S. de Brum, Luis F. O. Silva, Guilherme Luiz Dotto, Weslei Ambros, Eder C. Lima, Glaydson Simões dos Reis

**Affiliations:** a Xi'an Key Laboratory of Advanced Photo-electronics Materials and Energy Conversion Device, Xijing University Xi'an 710123 China; b Monte-Bianco Diamond Applications Co., Ltd. Foshan 528313 China; c School of Materials Science and Engineering, Xi'an University of Architecture and Technology Xi'an 710055 China; d Laboratory of Industrial Chemistry and Reaction Engineering, Faculty of Science and Engineering, Åbo Akademi University 20500 Åbo/Turku Finland glaydson.simoesdosreis@abo.fi; e Institute of Chemistry, Federal University of Rio Grande do Sul (UFRGS) Av. Bento Gonçalves 9500 Porto Alegre RS Brazil; f Universidad de La Costa, CUC Calle 58 # 55-66 Barranquilla Atlántico Colombia lfsoacademico@gmail.com; g Federal University of Santa Maria, Chemical Engineering Department Roraima Avenue, Postal Code 1000 97105-900 Santa Maria RS Brazil

## Abstract

Diclofenac sodium (DCF) is widely used and ends up in water bodies, posing a serious environmental threat. Carbon-based materials are effective adsorbents for removing contaminants from polluted waters. In this study, we synthesized monolayer molybdenum disulfide-doped carbon material (MoS_2_@CM) *via* a high-temperature process to assess its efficiency as an adsorbent for DCF adsorption from aqueous solution. The characterization data indicated that MoS_2_ doping significantly affected the physicochemical properties of the MoS_2_@CM structure. MoS_2_-nanoparticles enabled the formation of metal–sulfur–oxygen bonds, which boosted the adsorption of DCF molecules. The suitability of MoS_2_@CM as an adsorbent for DCF removal was investigated. The MoS_2_@CM exhibited much faster adsorption kinetics, with an adsorption capacity of 424 mg g^−1^ at 45 °C. Based on indirect evidence from pH effect, zeta potential, kinetic, and isotherm data, as well as literature on MoS_2_-doped carbons, we hypothesize that the following interactions may contribute to the adsorption of DCF onto MoS_2_@CM: pore filling, hydrogen bonding, π–π interactions, and possible electrostatic attractions. The adsorbents were subjected to regeneration, and the results indicated that both adsorbents could be reused for at least 5 consecutive cycles, with MoS_2_@CM exhibiting a higher reusability than @CM. The @CM and MoS_2_@CM adsorbents showed excellent performance in treating a synthetic effluent, with percentage removals of 78.7% and 80.2% of the total compounds, respectively, highlighting their potential for treating real effluents. This research provides new pathways for the preparation of biomass/metal/heteroatom materials for effective application in the removal of emerging contaminants from aqueous solutions, and beyond.

## Abstract 2

Diclofenac sodium adsorption on carbon/molybdenum disulfide porous surface.

## Graphical abstract

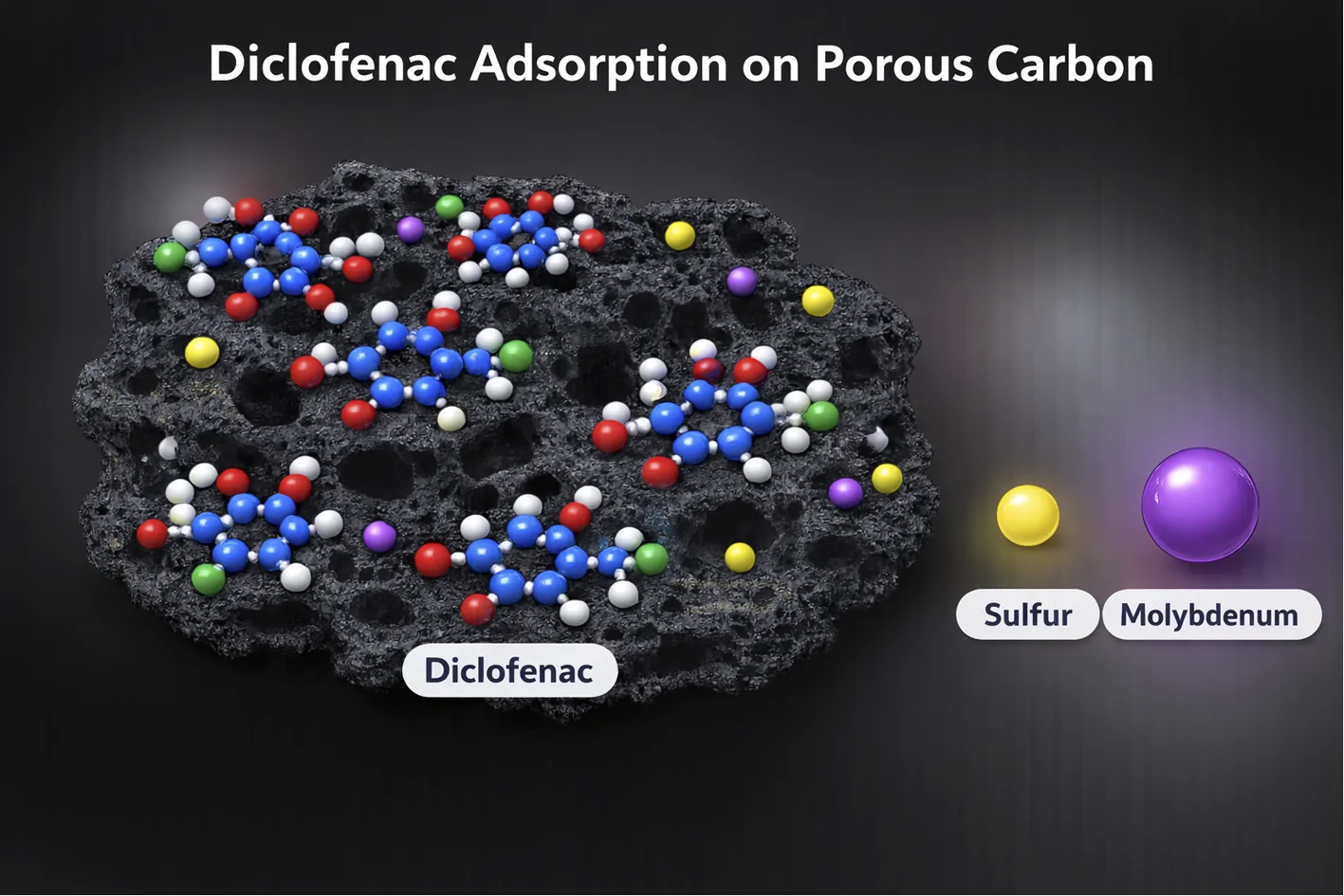

## 1. Introduction

The increasing contamination of water resources by human activities, particularly contaminants from pharmaceuticals, has attracted much attention over the last few decades.^1,2^ Pharmaceuticals are being detected in water resources all over the world due to their widespread use and improper disposal. Sodium diclofenac (DCF) is a popular pharmaceutical, a non-steroidal anti-inflammatory drug largely consumed every year, with an average consumption rate of approximately 0.33 ± 0.29 g per person per year.^3^ Without proper treatment, it can be discharged into water bodies, which poses a severe threat to aquatic ecosystems and human health. To address this issue, the development of efficient and sustainable technologies and materials to assist better removals of pharmaceuticals,^4^ including DCF from wastewaters, is welcome, and it has gained considerable attention in recent years.^5–7^

Among the various materials explored, biomass-derived carbon has been widely employed as an efficient and sustainable candidate adsorbent for the pharmaceutical uptake, due to its unique structural, chemical, and environmental properties.^8,9^ Biomass carbon, derived from renewable and abundant sources such as agricultural waste, forestry residues, and algae, offers a cost-effective and eco-friendly alternative to conventional adsorbents. Its high surface area, porous structure, and tunable surface chemistry make it an excellent material for adsorption applications.^10^ However, the adsorption capacity of pristine biomass carbon can be limited by its relatively low reactivity and selectivity. To overcome these limitations, the incorporation of MoS_2_, a two-dimensional transition metal dichalcogenide, has been proposed as an effective strategy to enhance the adsorption performance of biomass carbon.^11–13^

MoS_2_ is known for its layered structure, high surface area, and excellent chemical stability, which make it suitable for various environmental applications, including catalysis, energy storage, and pollutant removal.^14^ When doped into biomass carbon, MoS_2_ can provide additional active sites for organic molecules adsorption, improve the material's mechanical strength, and enhance its affinity for specific organic molecules. The synergistic effects between biomass carbon and MoS_2_ result in a composite material with superior adsorption capacity, faster kinetics, and better regeneration potential compared to its individual components.^15,16^

This paper explores the synthesis, characterization, and application of biomass carbon doped with MoS_2_ for pharmaceutical adsorption, along with the key factors influencing the material's properties and performance. The adsorption mechanisms, such as electrostatic interactions, hydrogen bonding, and π–π stacking, are also elucidated to provide a deeper understanding of the DCF removal process. Furthermore, the paper highlights the potential of this composite material for large-scale wastewater treatment and its environmental benefits, such as reduced carbon footprint and utilization of renewable resources. In conclusion, biomass carbon-doping with MoS_2_ represents a promising and sustainable solution for the removal of drugs from polluted wastewaters. Its unique combination of high adsorption capacity, cost-effectiveness, and environmental friendliness makes it a viable alternative to conventional adsorbents. Future research should focus on optimizing the synthesis process, exploring the adsorption of a wider range of pollutants, and evaluating the long-term stability and reusability of the material to facilitate its practical application in real-world scenarios.

## 2. Materials and methods

### 2.1. Materials

The DCF solutions were prepared with deionized water, and phosphoric acid (H_3_PO_4_), molybdenum disulfide (MoS_2_), and sodium diclofenac (DCF) were purchased from Sigma-Aldrich and used as received, with analytical grades specified by the sender.

### 2.2. Carbon material preparation

**Fig. 1:**
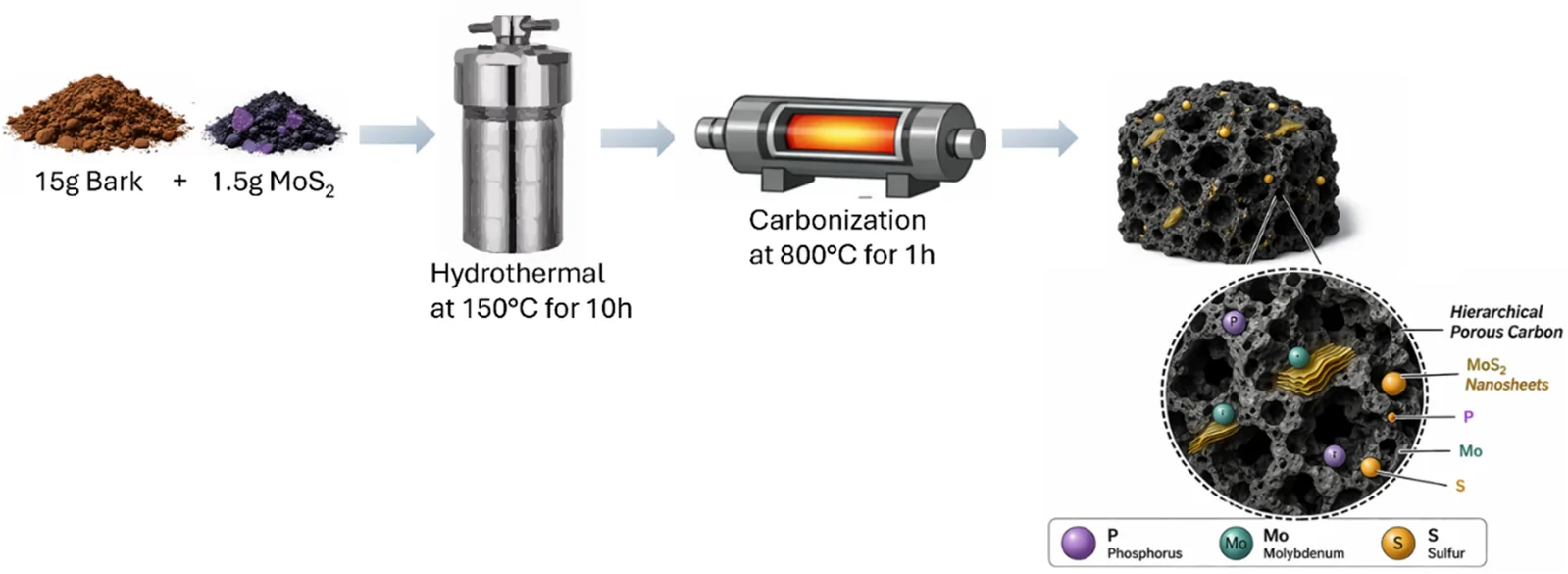
Schematic of the preparation steps of the MoS_2_@CM.

The carbon adsorbents were synthesized following the steps outlined in Fig. 1. Firstly, 15 g of the bark (particle size lower than 0.5 mm) were mixed with 1.5 g of MoS_2_, and added into the hydrothermal carbonization (HTC) vessel with no pressure control. 100 mL of H_3_PO_4_ 30% was added to the vessel and properly mixed. The vessel was heated at 150 °C for 10 hours. After heating, the sample was dried, placed in a ceramic crucible, and pyrolyzed at 800 °C for 1 hour (10 °C min^−1^). The pyrolysis oven was turned off and cooled at room temperature. After cooling, the prepared materials were washed with hot water until the pH of the washing water remained constant. Subsequently, the adsorbents were dried (105 °C) and named @CM (non-doped) and MoS_2_@CM (doped with MoS_2_).

### 2.3. Characterization of the adsorbent materials

Specific surface area (SSA) was obtained from N_2_ isotherms measured by a sorptometer (Tristar 3000, Micromeritics Instrument Corp., Norcross, GA, USA). The adsorbent materials' surfaces were analyzed using scanning electron microscopy (SEM). XPS analysis was performed using a Kratos Axis Ultra spectrometer. Raman spectra were obtained from a Bruker spectrometer. The amorphous/crystalline feature of both biochar materials was analyzed by a Rigaku SmartLab 9 kW X-ray diffractometer, using the International Center for Diffraction Data ICDD to identify the peaks. Transmission electron microscope (TEM) was employed to evaluate the @CM and MoS_2_@CM microstructures using a transmission electron microscopy (STEM; JEOL JEM-2200FS EFTEM/STEM). The samples were dispersed in ethanol, sonicated for 1 minute, and a drop of the solution was manually deposited onto a carbon-coated copper grid.

### 2.4. Adsorption studies

Batch adsorption was employed to test the adsorption capacity of @CM and MoS_2_@CM for DCF from aqueous solutions. The effects of pH, contact time, and initial DCF concentration were studied. All adsorption experiments were performed in 50.0 mL Falcon tubes containing 20 mL of solution and approximately 30 mg of adsorbent (1.5 g L^−1^). The pH was varied (from 2 to 10) to study its effect on DCF removal. A kinetic was performed using a fixed DCF solution concentration of 250 mg L^−1^ and 1.5 g L^−1^ of adsorbent dosage, with a contact time from 0 to 360 min. For isotherm experiments, the initial DCF concentration ranged from 50 to 1200 mg L^−1^. All experiments were conducted at a shaking speed of 250 rpm. The remaining DCF solutions were detected by UV-visible spectrophotometry at 276 nm. The removal of DCF was calculated from where it was determined based on eqn (1) and (2), respectively, described in the SI.

### 2.5. Effluent treatment

An effluent loaded with several drugs and organic/inorganic compounds was prepared to simulate realistic hospital effluents and test the @CM and MoS_2_@CM abilities to treat real wastewaters. The composition of the synthetic effluent is shown in the SI (Table S1).

## 3. Results and discussion

### 3.1. Physicochemical properties of @CM and MoS_2_@CM adsorbents

**Fig. 2:**
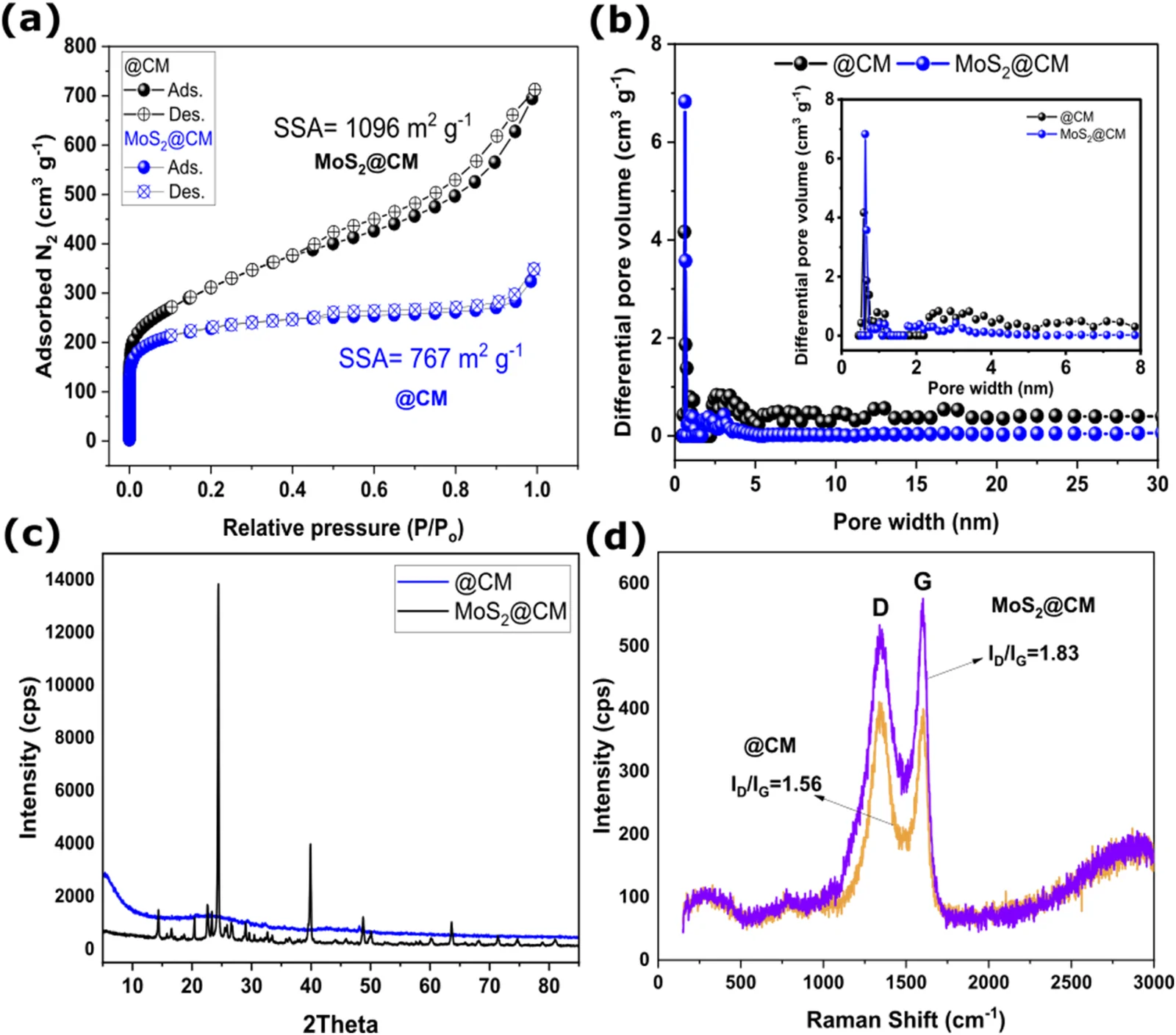
(a) N_2_ isotherm for @CM and MoS_2_@CM, (b) pore distribution curves for @CM and MoS_2_@CM, (c) XRD diffractograms for @CM and MoS_2_@CM samples, (d) Raman spectra for @CM and MoS_2_@CM samples.

The porosity of @CM and MoS_2_@CM was determined by N_2_ isotherms (see Fig. 2(a and b)). From N_2_ isotherms, the specific surface area (SSA) can be obtained. SSA plays an essential role in the adsorption ability of an adsorbent. For both materials, N_2_ isotherms indicate very high porosity. However, important differences are highlighted, confirming the impact of MoS_2_ on the MoS_2_@CM porosity features. Based on the IUPAC classification, MoS_2_@CM exhibited isotherm shapes typical of types I and IV. Isotherm type I indicates the presence of micropores in its structure, as evidenced by the high N_2_ adsorption at lower pressures, while type IV is characteristic of mesoporosity due to the hysteresis loop.^17^

The SSA values for @CM and MoS_2_@CM are 1096 and 767 m^2^ g^−1^, respectively. It is seen that the treatment with MoS_2_ provoked a reduction in the SSA value, which could be due to the collapse and enlargement of micropores by leading to the enhancement of the mesoporosity; at the same time, Mo and sulfur atoms can stick into the pores of the carbon, which reduces the area available in the porous structure. However, both samples exhibited high SSA, which could positively influence the removal of DCF due to its molecular size, which easily fits within the mesopores.

To corroborate the N_2_ curves discussion, the pore size distribution curves of carbon materials are exhibited in Fig. 2(b). Both curves show large portions of micropores at diameters of around 1 nm. In addition, small mesopores are observed in the region of 2–4 nm (see inset in Fig. 2(b)). Also, for @CM, some portions of mesopores 17 nm are also observed. As demonstrated in the N_2_ isotherms, both carbons have a great combined presence of micro-mesopores in their structures.

XRD analysis was employed to investigate the crystalline or amorphous features of the @CM and MoS_2_@CM adsorbent materials (Fig. 2(c)). The XRD pattern for @CM indicates a total amorphous structure, typical of biomass-based carbon structures. On the other hand, MoS_2_@CM displayed a totally different structure, highly crystalline, highlighting the successful modification of @CM by MoS_2_. Many sharp peaks at 14.3, 20.4, 22.5, 23.3, 24.4, 33.4, 39.1, 48.7, 63.7° planes of well-crystalline peaks, which are MoS_2_ with a hexagonal phase (JCPDS: 06-0097), respectively. Besides these sharpers, many other small peaks are observed throughout the diffractogram, highlighting its successful modification by MoS_2_.^18,19^

The Raman technique was employed to evaluate further the microstructure of the @CM and MoS_2_@CM adsorbent materials, focusing on their degree of order/disorder/graphitization (see Fig. 2(d)). Two distinct peaks are observed at around 1343 cm^−1^ and 1605 cm^−1^, which are related to the carbon lattice defects (and disordered structures in amorphous carbon) and graphitic crystalline structures (C atoms with sp^2^ electronic configuration), respectively.^20,21^ From Raman, *I*_D_/*I*_G_ was obtained for the two samples, which was used to indicate the level of defects and graphitization. The *I*_D_/*I*_G_ values of @CM and MoS_2_@CM were 1.56 and 1.83, respectively. This suggests that MoS_2_ incorporation led to more disordered, defective structures in the MoS_2_@CM carbon lattice.^20,21^ It is reported that defects in carbon structures can serve as additional adsorption-active sites for adsorbing molecules/ions; thus, incorporating MoS_2_ into carbonaceous materials is an effective strategy to produce high-performance adsorbent materials.

**Fig. 3:**
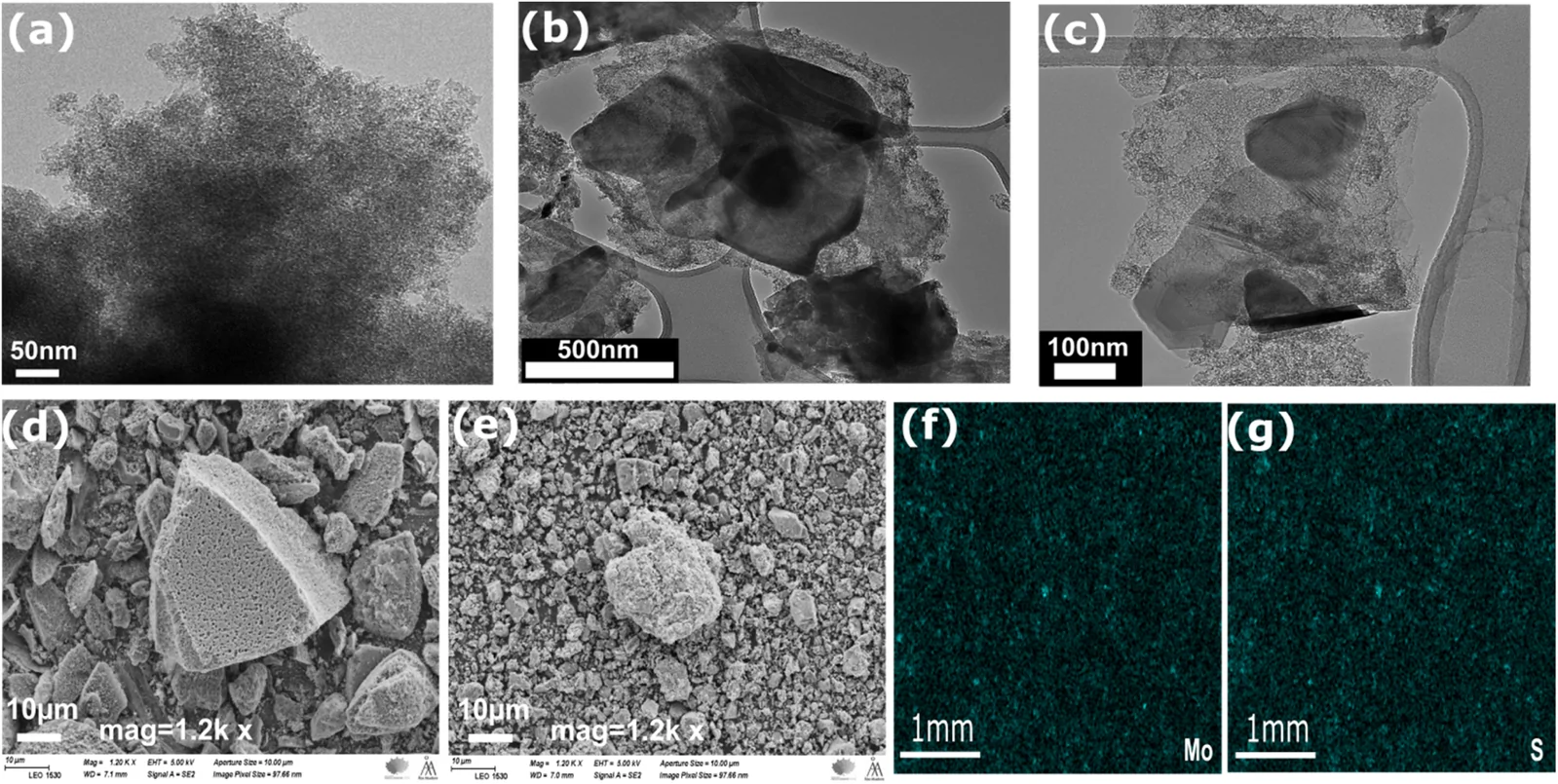
TEM images of (a) @CM and (b and c) MoS_2_@CM samples, SEM image of (d) @CM and (e) MoS_2_@CM samples, and mapping of the elements (f) molybdenum and (g) sulfur for the MoS_2_@CM sample.

The effect of MoS_2_ doping was further evaluated by TEM. As shown in Fig. 3(a), @CM shows a predominant amorphous structure. Fig. 3(b) and (c) shows the MoS_2_@CM structure, whereas the MoS_2_ layered structure is visible over the amorphous structure of the carbon matrix. It seems that the MoS_2_ is mixed with amorphous carbon. To further evaluate the presence of MoS_2_ in the MoS_2_@CM structure, STEM-EDX mapping is shown in Fig. S1 (mapping of the elements molybdenum and sulfur). The figure shows that both elements are mostly homogeneously distributed throughout the carbon structure, indicating that the @CM was successfully modified by MoS_2_ incorporation.

SEM images of the samples are shown in Fig. 3(d and e). The images show important morphological differences; for instance, @CM sample denser structures full of small visible holes (see Fig. S2), on the other hand, MoS_2_@CM exhibits a more broken and irregular/rougher structures as shown in Fig. 3(e) and S3, which indicate that the doping process with MoS_2_ successfully modified the physical/morphological appearance of the material, which could be beneficial for adsorbing organic molecules.

**Fig. 4:**
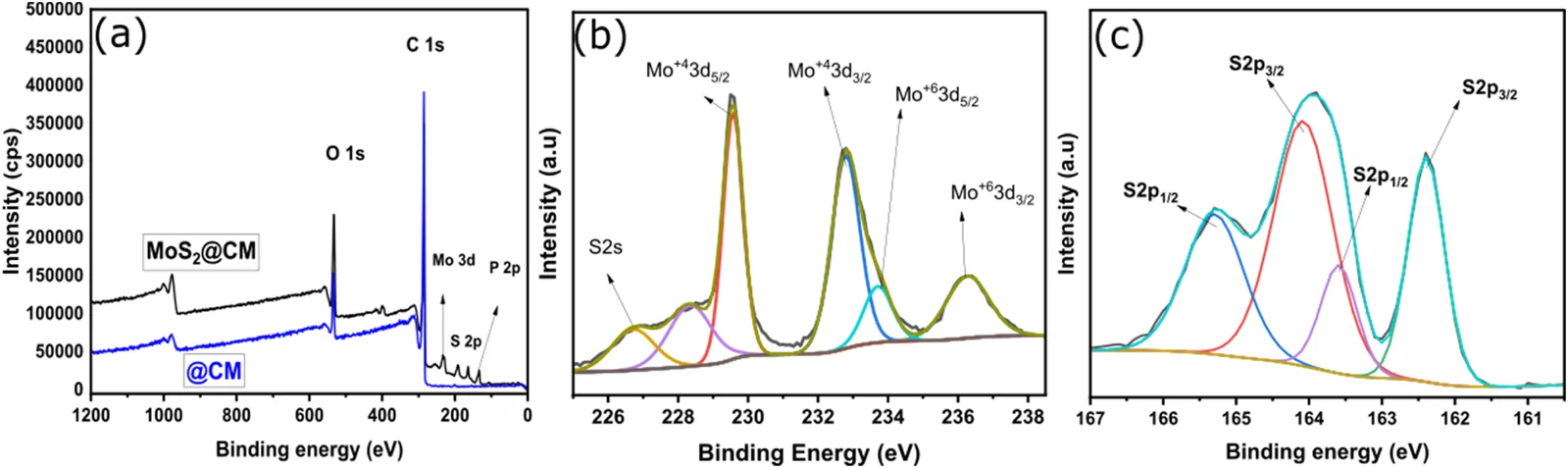
(a) Survey XPS spectra for @CM and MoS_2_@CM samples, (b) high-resolution Mo 3d XPS spectra of MoS_2_@CM, and (c) high-resolution S 2p XPS spectra of MoS_2_@CM.

XPS analysis was employed to explore the materials' surface element chemical information before and after MoS_2_ modification (Fig. 4). The survey spectra (Fig. 4) exhibit an evident difference between the two samples, highlighting the successful modification of @CM by MoS_2_. The survey spectrum for MoS_2_@CM shows sharp peaks in the Mo 3d and S 2p regions (see Fig. 4(a)), confirming its successful modification by MoS_2_ doping. For deeper evaluation of the doping process, the Mo spectrum was deconvoluted, and the results show that the molybdenum (Mo 3d) spectrum was deconvoluted into four peaks (Fig. 4(b)), centered at 236.2 and 231.8 eV, which could be related to Mo 3d_3/2_ and 3d_5/2_ from MoO_2_, respectively.^22^ The additional peaks at 231.7 and 229.7 eV could be assigned to Mo 3d_3/2_ and 3d_5/2_ from MoS_2_.

The existence of Mo^6+^ can be attributed to the oxidation of partial Mo^4+^ during the synthesis of MoS_2_.^23^Fig. 4(c) displays the high-resolution spectrum of sulfur (S 2p), with four peaks, suggesting that the MoS_2_@CM contains different sulfur states and, once again, highlights the successful modification of the @CM material.

### 3.2. Adsorption experiments of DCF on @CM and MoS_2_@CM adsorbents

#### 3.2.1 Adsorbent's mass and temperature effect on DCF removal

**Fig. 5:**
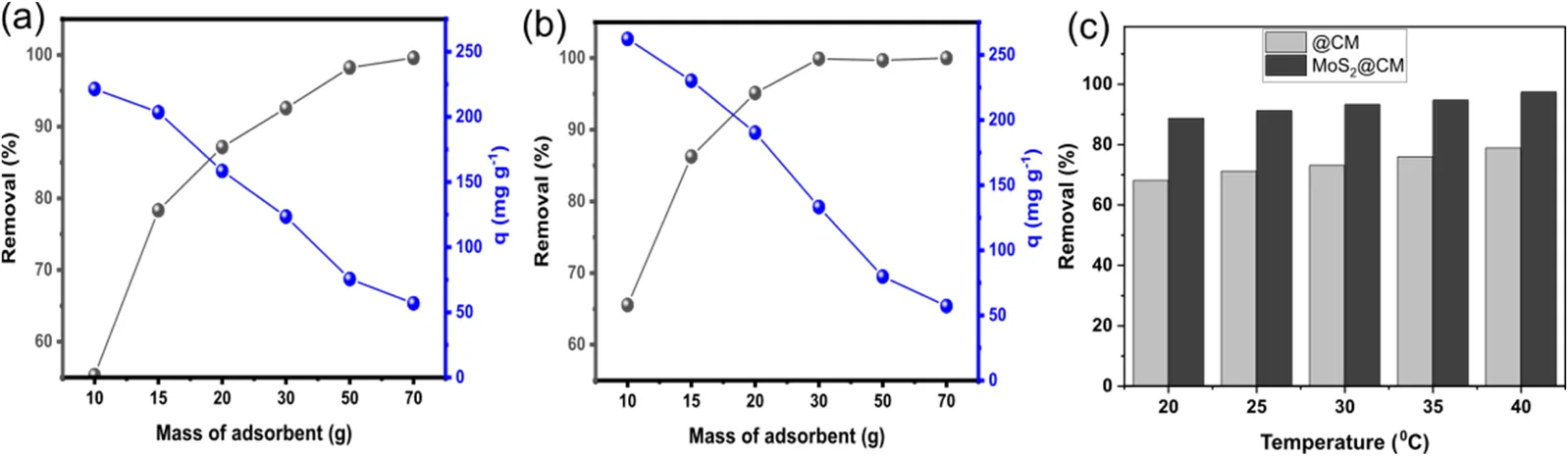
(a) Mass effect on DCF removal for @CM sample, (b) mass effect on DCF removal for MoS_2_@CM sample, (c) temperature effect on DCF removal for @CM and MoS_2_@CM samples.

In practical terms, the adsorbent's mass is of great importance because it impacts the costs and the scalability process of an adsorption system. Fig. 5(a and b) exhibits the adsorbent's mass effect on DCF removal. Both samples show similar curves. The DCF removal increases as both adsorbents' masses increase, while their adsorption capacities (*q*) decrease. The rapid increase in DCF adsorption as the adsorbent mass increases is associated with a large number of adsorption-active sites accessible to adsorbing DCF molecules.

The mass effect varied the adsorbent mass amount from 20 to 70 mg. The figure displays that MoS_2_@CM presented higher performance than @CM. This can be explained by its high SSA and abundant adsorption sites due to the MoS_2_ incorporation on the carbon matrix. The better adsorbent (MoS_2_@CM) was employed to evaluate the optimal adsorbent mass. The optimal adsorbent mass was chosen to be 30 mg (1.5 L g^−1^ of adsorbent dosage). This selection is based on the fact that at this dosage, 99.8% of the DCF was removed from the solution, with an initial concentration of 200 mg L^−1^. Therefore, the next experiments (temperature effect, pH effect, kinetics, and isotherm) were carried out using 1.5 g L^−1^ of adsorbent dosage.

The effect of temperature on DCF removal was also investigated (see Fig. 5(c)). The temperature varied from 20 °C to 40 °C. It is seen that the temperature slightly influenced DCF removal by both adsorbents. For @CM, at 20 °C, the removal was 68.1% and increased to 78.9% at 40 °C. For MoS_2_@CM, at 20 °C, the removal was 88.7% and increased to 97.4% at 40 °C. In an adsorption system, the increase in DCF removal with increasing temperature indicates that adsorption of DCF onto both adsorbents (@CM and MoS_2_@CM) is endothermic. Although the temperature did influence DCF removal, its effect was not significant. Thus, we fixed the temperature of 25 °C for the next experiments. An increase from 25 °C certainly implies very high operational costs, which do not justify it.

#### 3.2.2 Zeta potential and pH effect

**Fig. 6:**
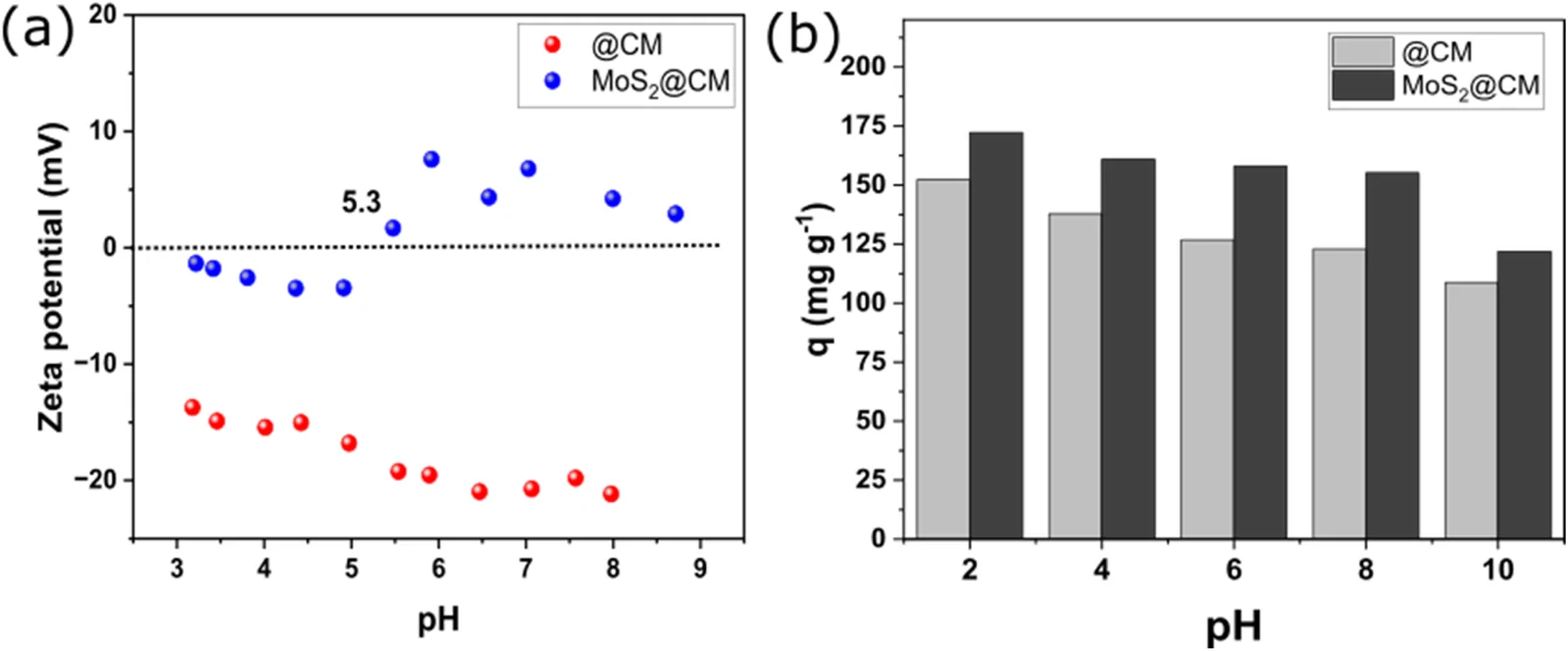
(a) Zeta potential and pH_pzc_, and (b) the effect of pH on DCF adsorption.

The pH of the point of zero charges (pH_pzc_) of @CM and MoS_2_@CM adsorbents was obtained from zeta potential measurements (see Fig. 6(a)). The pH_pzc_ is of crucial interest because it indicates at which pH the surface of the adsorbent is neutral, as well as indicates at which pH the surface of the adsorbent is either positively or negatively charged.

Fig. 6(a) shows that the zeta potential of @CM remains negative across different pH values, highlighting its negatively charged surface. A more negative surface charge tends to increase the electrostatic repulsion among @CM and DCF because DCF is an anion, thereby hindering the adsorption. However, MoS_2_@CM exhibited a pH_pzc_ of 5.3, meaning that above it the surface becomes positively charged. This facilitates the adsorption process due to electrostatic attraction between MoS_2_@CM and DCF. Mo tends to lose electrons, forming cations with common oxidation states of +4 and +6,^24^ which could have influenced the positively charged surface of MoS_2_@CM.

Fig. 6(b) shows how the pH affects the ability of the @CM and MoS_2_@CM in adsorbing DCF molecules. The results prove that the pH indeed affected the amount of DCF adsorbed by the materials. The DCF removal slightly increased with the pH decreasing from 2 to 10. The poorer DCF removal at higher pH could be related to the competition of excessive OH^−^ for being attracted by active sorption sites. Thus, it can be said that electrostatic attraction also contributes to the adsorption of DCF on @CM and MoS_2_@CM adsorbents. It is observed that acidic conditions favored DCF adsorption. DCF molecular form exists when the pH is lower than its p*K*_a_ value (4.35 ± 0.2), while its ionic form is predominant at pH values higher than the p*K*_a_ value.^25–27^ At pH levels lower than the p*K*_a_, the carboxylic acid group of DCF is protonated, resulting in a neutral molecule, which displays reduced electrostatic repulsion, making it more prone to adsorption onto surfaces.

#### 3.2.3 Kinetic and equilibrium studies for DCF adsorption

**Fig. 7:**
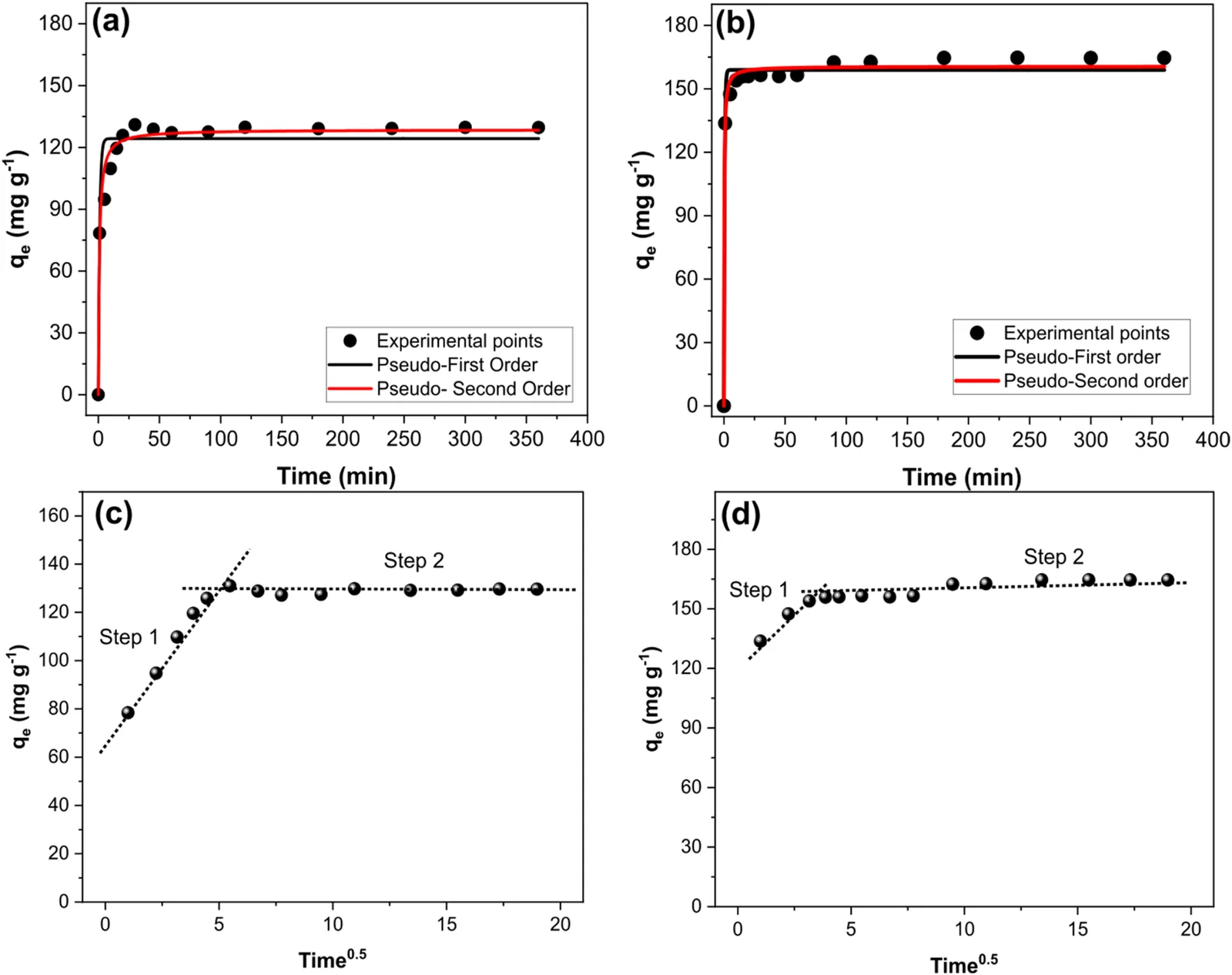
Kinetic measurements for DCF adsorption of (a) @CM, (b) MoS_2_@CM; and equilibrium curves for DCF adsorption of (c) @CM, (d) MoS_2_@CM.

The kinetics in an adsorption system are vital because they provide insights into the adsorption process between an adsorbate and an adsorbent material.^28,29^Fig. 7(a and b) shows the fitted curves for the kinetics of DCF on CM and MoS_2_@CM adsorbents. The plots exhibit different behaviors, showing that the MoS_2_@CM adsorbent was much faster, adsorbing 133 mg g^−1^ in the first minute, while @CM adsorbed only 78 mg g^−1^; a similar trend was observed by Yin *et al.*,^12^ which employed an adsorbent based on MoS_2_-carbon composite for dye removal, and the large majority of the adsorbed molecules were during the first minute. This improved the adsorptive performance, which could be due to the presence of more surface functionalities on the MoS_2_@CM adsorbent sample, which acts as active adsorption sites to boost its adsorptive properties.^30^

For a deeper evaluation of the kinetic processes, the kinetic data were evaluated using the pseudo-first-order and pseudo-second-order non-linear kinetic models. The suitability of these models was evaluated by taking into account *R*_adj_^2^ and SD values. A higher *R*_adj_^2^ and lower SD values indicate a smaller disparity between the *q* measured experimentally and the theoretical *q* values obtained by the models. Based on that, the pseudo-second order model presented better suitability for both adsorbents due to its highest *R*_adj_^2^ and lowest SD values (see Table 1).

**Table 1:** Kinetic and equilibrium parameters for DCF adsorption

	@CM	MoS_2_@CM
**Pseudo-first-order**		
*q*_e_ (mg g^−1^)	124.3	158.9
*k*_1_ (min^−1^)	0.921	1.838
*R*_adj_^2^	0.919	0.9843
SD (mg g^−1^)	9.816	5.177

**Pseudo-second-order**		
*q*_e_ (mg g^−1^)	128.6	160.5
*k*_2_ (g mg^−1^ min^−1^)	0.00906	0.2756
*R*_adj_^2^	0.9714	0.9918
SD (mg g^−1^)	5.868	3.742

To further understand the kinetic process of DCF over @CM and MoS_2_@CM, the intraparticle diffusion was employed to fit the kinetic data (see Fig. 7(c) and (d)). The DCF adsorption on carbon-based materials often faces a multi-step process, where DCF molecules are transported from the solution to the surface of the @carbon materials, followed by the diffusion to the inner pores into @CM and MoS_2_@CM. Both adsorbents exhibit two stages of the rate of adsorption, with the first stage (step 1), which could be attributed to the boundary diffusion and intraparticle diffusion into the adsorbent pores. This step is usually the fastest, and comparing the adsorbents, it is faster for MoS_2_@CM than for @CM, possibly because MoS_2_@CM offers less resistance and better adsorption capacity due to the additional adsorption sites provided by MoS_2_ incorporation. Stage 2 (step 2) can be related to diffusion from the surface through smaller pores until equilibrium is attained.

**Fig. 8:**
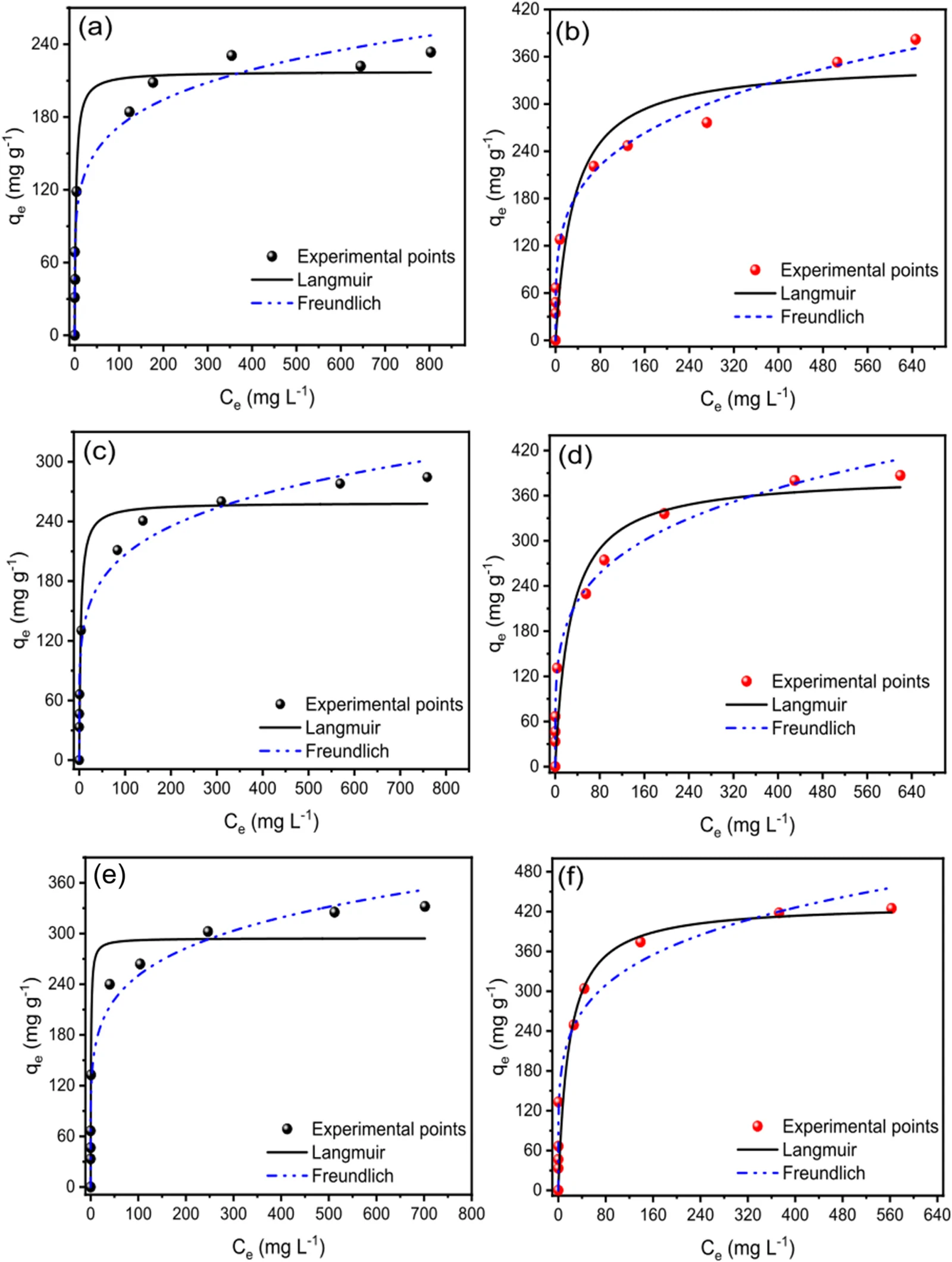
Equilibrium curves for DCF adsorption for 25 °C (a) @CM, (b) MoS_2_@CM, for 35 °C (c) @CM, (d) MoS_2_@CM, and 45 °C (e) @CM, (f) MoS_2_@CM.

The equilibrium isotherms of DCF adsorption for @CM and MoS_2_@CM at different temperatures (25 °C, 35 °C, and 45 °C) are shown in Fig. 8(a–f). The experimental data were fitted by the Langmuir and the Freundlich nonlinear models. As the kinetics, the equilibrium model's suitability was evaluated taking into account their *R*_adj_^2^ and SD values. Based on Table 2, the values of these parameters indicate that Freundlich presented the best fitted values for both adsorbents, which suggest a more heterogeneous adsorption process, and a high affinity between DCF and adsorbents: however, is clear that MoS_2_@CM showed a much higher affinity in adsorbing DCF molecules due to its total removal of DCF in low concentration and higher adsorption amounts at higher initial DCF concentrations, due to the large abundance in adsorption active sites on MoS_2_@CM for adsorbing DCF. Regarding the effect of the temperature on the isotherms, it is reported that the DCF is strongly influenced by temperature,^31^ which suggests that the DCF removal increases with the increase of the temperature that positively affects the sorption rate due to the sorbate diffusion enhancing^32^ The isotherm studies under different temperatures are in accordance with the preliminary studies reported in Fig. 4(c).

Isotherm parameters for the adsorption of DCF on @CM and MoS_2_@CM adsorbents

**Table:** 

@CM	298 K	308 K	318 K
**Langmuir**			
*q*_max_ (mg g^−1^)	218	259	294
*K*_L_ (L min^−1^)	0.35	0.30	1.21
*R*_adj_^2^	0.9295	0.9434	0.9471
SD (mg g^−1^)	24.4	26.6	30.3

**Freundlich**			
*K*_F_ ((mg g^−1^) (mg L^−1^)^−1/*n*_F_^)	77.3	87.7	111.9
*n*_F_	5.75	5.37	5.72
*R*_adj_^2^	0.9609	0.9817	0.9750
SD (mg g^−1^)	18.1	15.1	20.8

**Table:** 

MoS_2_@CM	298 K	308 K	318 K
**Langmuir**			
*q*_max_ (mg g^−1^)	353	387	432
*K*_L_ (L min^−1^)	0.031	0.037	0.056
*R*_adj_^2^	0.8925	0.9086	0.8983
SD (mg g^−1^)	45.4	45.6	53.7

**Freundlich**			
*K*_F_ ((mg g^−1^) (mg L^−1^)^−1/*n*_F_^)	75.8	95.2	129
*n*_F_	4.08	4.41	5.02
*R*_adj_^2^	0.9937	0.9914	0.9715
SD (mg g^−1^)	11.0	14.0	28.4

From the Freundlich model, important information can be extracted, such as the *K*_F_ value, which represents the adsorption capacity/affinity of an adsorbent for a solute.^33,34^ A higher *K*_F_ value generally indicates a greater, stronger, and more favorable adsorption capacity, and the *K*_F_ increased with the temperature increasing,^34^ suggesting the DCF adsorption affinity towards @CM and MoS_2_@CM adsorbents was higher at 45 °C.

Although the effect of temperature on the adsorption capacity was evaluated, a formal thermodynamic assessment, specifically the determination of the standard enthalpy (Δ*H*°) and entropy (Δ*S*°) of adsorption, could not be reliably conducted. As observed in Table 2, the Langmuir affinity constants (*K*_L_) vary non-uniformly with temperature, lacking a consistent mathematical trend. Upon converting the *K*_L_ values to standard dimensionless thermodynamic equilibrium constants (*K*^0^_e_), the data did not exhibit a clear correlation with temperature. Consequently, it was not possible to obtain a valid mathematical fit using either the classical linear van't Hoff equation (ln *K*^0^_e_*versus* 1/*T*) or the non-linear van't Hoff equation (*K*^0^_e_*versus T*). The application of thermodynamic models to a limited dataset of three temperatures, with no clear trend, yields mathematically forced parameters devoid of physical meaning. This deviation from classical van't Hoff behavior indicates that the adsorption enthalpy is not constant over the evaluated temperature range (298–318 K), which is consistent with the complex, multi-step interaction mechanisms (pore filling, hydrogen bonding, and π–π interactions) governing the adsorption of DCF onto the heterogeneous surface of the MoS_2_@CM composite.

#### 3.2.4 Comparison with literature

The kinetics and equilibrium adsorption data demonstrated that @CM and MoS_2_@CM adsorbents showed excellent affinity in adsorbing DCF from aqueous solutions. Thus, to obtain a better accessibility about their effectiveness, Table 3 is presented to compare @CM and MoS_2_@CM adsorption capacities with the most recent literature.^34–41^ For an easy comparison, we assume that the *q*_max_ values were obtained under optimum adsorption conditions. Therefore, MoS_2_@CM shows to be very effective in removing DCF from aqueous solutions, since it presented the second highest *q*_max_ value.

**Table 3:** Comparison of the *q*_max_ for DCF on different adsorbents

Adsorbents	*q*_max_ (mg g^1^)	Isotherm model	*T* (°C)	pH	Adsorbent dosage (g L^−1^)	Ref.
MnFe_2_O_4_ functionalized magnetic sawdust-based biochar	344.26	Sips	25 °C	4.0	0.15	35
Aquatic plant-derived biochars	23.25	Langmuir	20 °C	6.0	0.12	36
Chitosan/microcrystalline cellulose@polyethyleneimine hydrogel beads	274.84	Freundlich	25 °C	6.0	0.3	37
Multinetwork aerogels combined with UiO-66	775.9	Dubinin–Radushkevich	35 °C	7.0	0.56	38
Biochar from waste sludge/leaf	287.81	Temkin	25 °C	6.5	6.25	39
MgAl/layered double hydroxide supported on *Syagrus coronata* biochar	168.04	Sips	60 °C	5.0	0.05	40
Hierarchical ultra microporous aluminum based metal–organic aerogels	416	Dubinin–Radushkevich	25 °C	3.0	0.2	41
Lignin-based porous microspheres	426.18	Dubinin–Radushkevich	30 °C	7.0	0.133	42
UiO-66–NH_2_-based Zr-MOF composites	385	Langmuir	22 °C	7.0	0.25	43
Al(iii)-based MOFs (MOF-303)	334.89	Temkin model	∼30 °C	7.0	1.0	44
Al(iii)-based MOFs (DUT-5)	103.36	Temkin model	∼30 °C	7.0	1.0	44
Magnetic MOF adsorbent (DLU-1)	387.5	Langmuir	25 °C	6.53	0.2	45
Commercial activated carbon (F-300)	147.88	Langmuir	40 °C	6.0	4.0	46
@CM	336	Experimental	45 °C	6.0	1.5	This work
MoS_2_@CM	424	Experimental	45 °C	6.0	1.5	This work

Further evaluating the results in Table 3, it is observed that the adsorbent Multinetwork aerogels combined with UiO-66 presented a higher *q*_max_ than MoS_2_@CM; however, despite having a higher *q*_max_, it is important to point out that Multinetwork aerogels combined with UiO-66 presents a more complex preparation method than our adsorbent, which may suggest the expenditure of higher costs, not justifying its real utilization in treating wastewaters. On the other hand, MoS_2_@CM is prepared from an abundant and cheap, and therefore, sustainable source with potential for real application.

#### 3.2.5 Regeneration studies

**Fig. 9:**
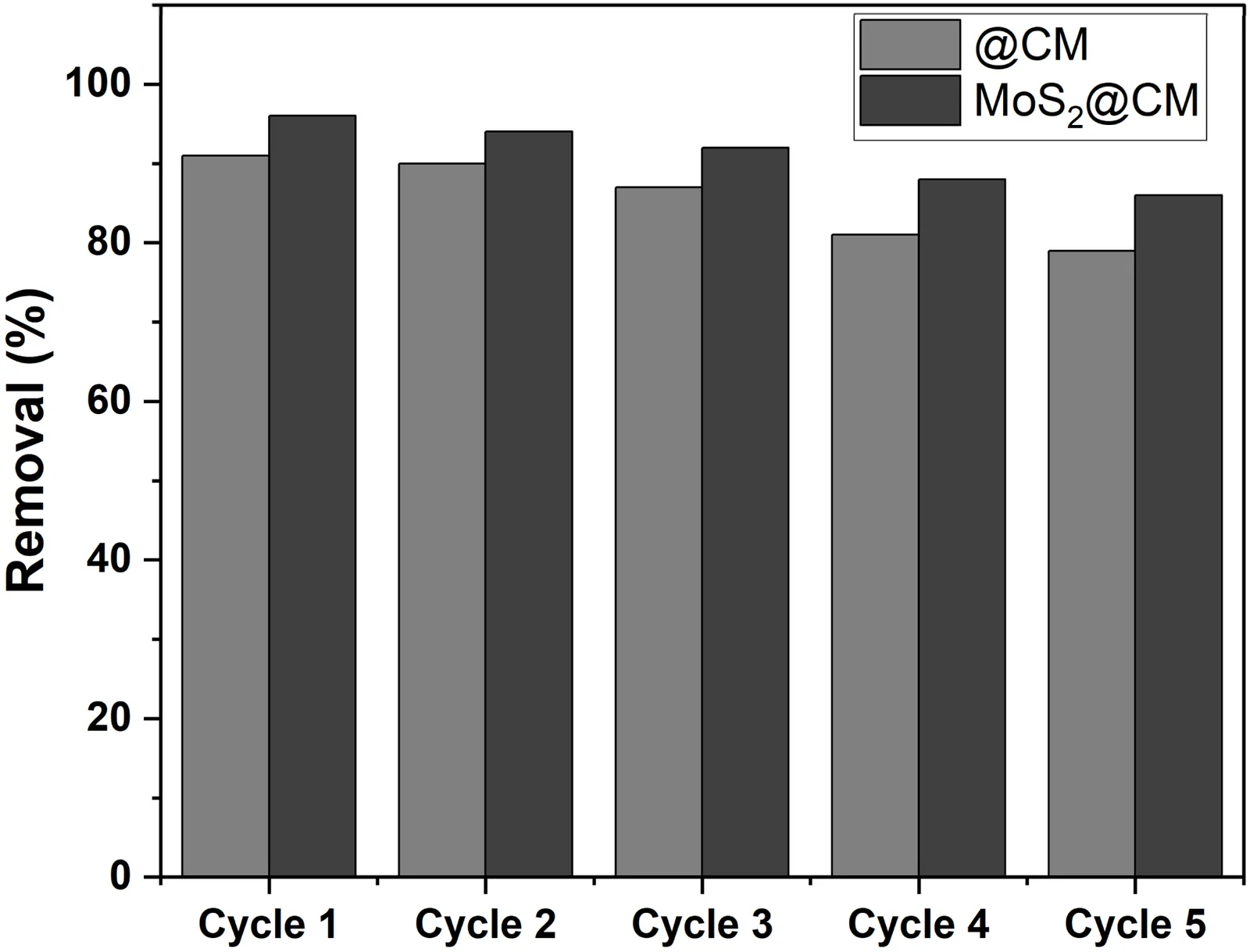
Regeneration studies for DCF's adsorption on @CM and MoS_2_@CM.

The adsorbent's reusability is a crucial step to evaluating its cost-effectiveness and suitability for the application in real adsorption systems. The reusability of the @CM and MoS_2_@CM adsorbents was examined to assess their performance effectiveness in being used multiple times (multiple adsorption–desorption cycles) (see Fig. 9). 0.1 M NaOH + 20% EtOH eluent was employed as the desorbing agent (Fig. 9). Both adsorbents displayed robust regeneration capacity, maintaining elevated removals after consecutive cycles, displaying a small decrease in performance. Comparing both samples, MoS_2_@CM exhibited better regeneration performance than @CM; after five cycles MoS_2_@CM was able to adsorb 86% while @CM adsorbed 79%. The small decrease in adsorption over the cycles could be due to DCF molecules adsorbed on both samples not being fully removed during the desorption step, which negatively impacts subsequent cycles. Some DCF molecules could be trapped in the pore network of the adsorbents, which reduces the available SSA to adsorb DCF in the next cycles. However, MoS_2_@CM adsorbent material maintained a good reusability degree after five cycles, which highlights its potential application in real systems.

#### 3.2.6 Adsorption mechanisms

**Fig. 10:**
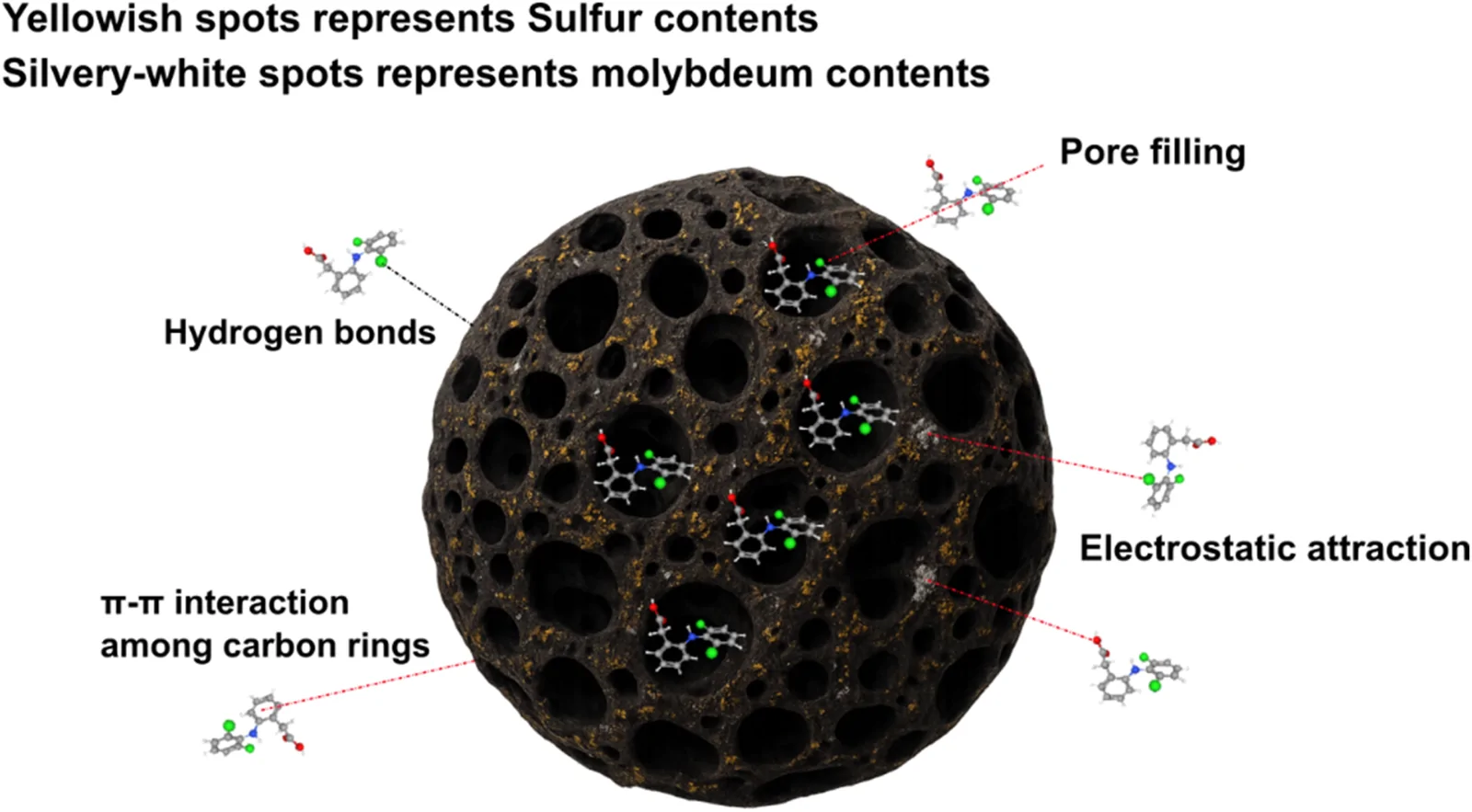
Proposed mechanism for DCF's adsorption onto MoS_2_@CM.

Based on the adsorption performance of the MoS_2_@CM adsorbent for DCF under various influencing factors, the mechanism of DCF removal by MoS_2_@CM is proposed, using indirect evidence, and involves multiple interactions, as demonstrated in Fig. 10. SEM, TEM, and XPS results indicate that the MoS_2_@CM structure contains sulfur-based and Mo-based groups that serve as abundant active adsorption sites capable of binding DCF molecules. The prepared MoS_2_@CM exhibited a porous structure with S and Mo distributed across its surface, facilitating the adsorption and diffusion of DCF molecules. Since the material has a high surface area (1009 m^2^ g^−1^), pore filling is one of the main mechanisms involved in the process.^4^

Hydrogen bonding and π–π interactions possess a contribution to the overall adsorption capacity. π–π interactions take place between the aromatic rings of DCF and the MoS_2_ layers, as well as the aromatic rings of the carbon matrix.^47^ Since the pH did not largely affect the adsorption of the DCF molecules, the electrostatic interactions have not played a significant role in the process. H-bonding takes place between atoms (*e.g.*, O, H, S) present in MoS_2_@CM and DCF molecules that form H bonds.^48,49^

The insertion of Mo-nanoparticles over the adsorbent's surface may interact with oxygen-based groups on the MoS_2_@CM's surface, which may form Mo–oxygen bond, creating structural defects (physicochemical and electronic defects) on the adsorbent's surface, which results in more active adsorption sites to boost the MoS_2_@CM's adsorptive properties (coordination bonding between diclofenac carboxylate groups and MoS_2_ active sites).^41,50^

#### 3.2.7 Synthetic effluent treatment

**Fig. 11:**
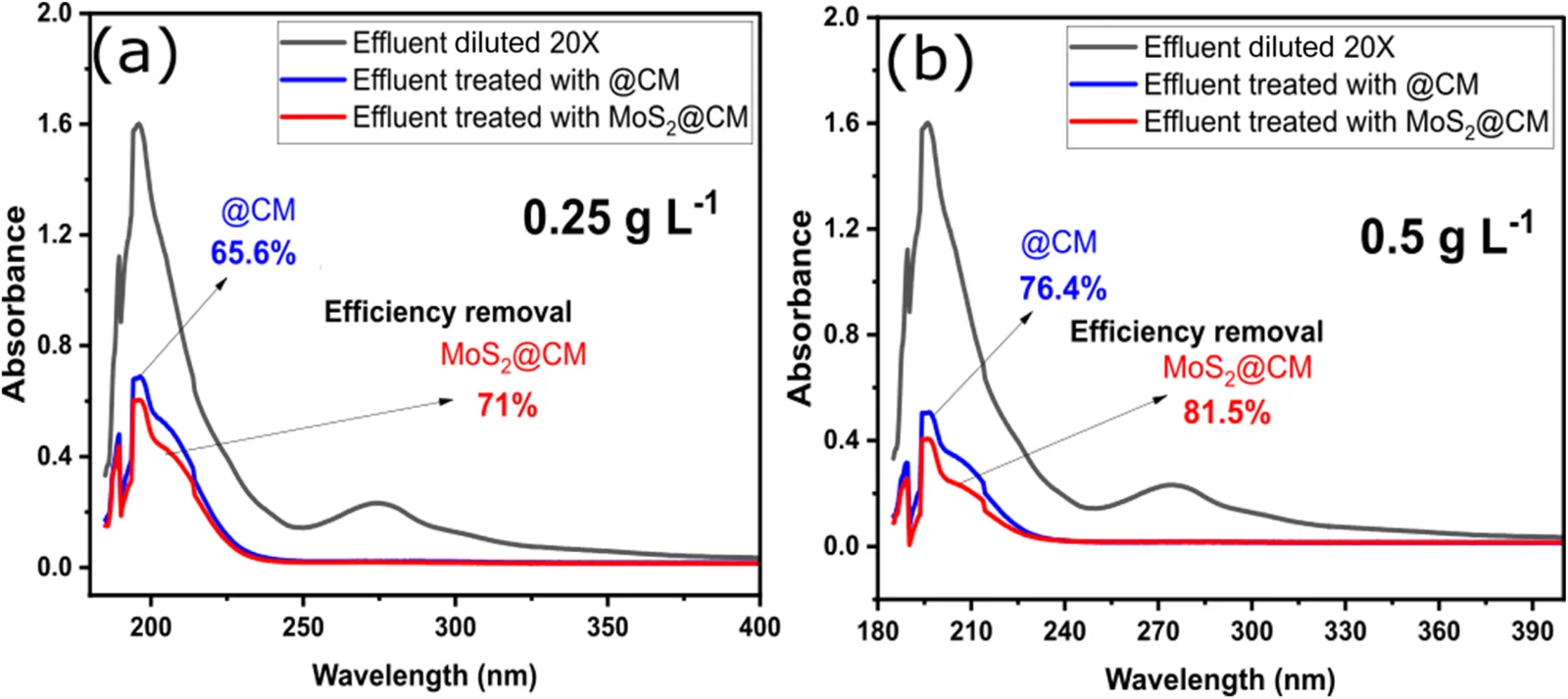
Effluent spectra of @CM and MoS_2_@CM materials for a low-concentrated effluent (Table S1 diluted 20 times) and different adsorbent dosages (a) 0.25 g L^−1^ and (b) 0.5 g L^−1^.

Since @CM and MoS_2_@CM demonstrated excellent removal of DCF from aqueous solutions, they were also employed in the removal of a synthetic effluent containing several pharmaceuticals, organic and inorganic compounds (simulating real and complex effluent, see Table S1), to evaluate their efficiency to clean such effluent. The amount of removal of the compounds in the effluent was calculated taking into account the areas under the UV-vis spectra from 190 to 400 nm of the synthetic effluent before and after the treatment^51^ (see Fig. 11 and S4).

Fig. 11(a and b) shows the spectra curves for an effluent at a much lower concentration than that of Table S1 (diluted 20 times), which is necessary to better reflect more realistic contamination levels in water bodies. For this low-concentration effluent, two adsorbent dosages were used (0.25 g L^−1^ and 0.5 g L^−1^), as shown in Fig. 11(a) and (b), respectively.

For the adsorbent dosage of 0.25 g L^−1^, the adsorbents exhibited excellent performance in removing 65.6% and 71% for @CM and MoS_2_@CM, respectively. At the adsorbent dosage of 0.5 g L^−1^, the removal increased to 76.4% and 81.5% for @CM and MoS_2_@CM, respectively. Such results indicate that our adsorbents have great potential for removing drugs in synthetic effluents.

The materials demonstrated good ability in treating low-concentration effluents; however, they were also employed in the removal of a highly concentrated effluent (see Table S1). The spectra curves show that MoS_2_@CM presented excellent percentage removal (80.2%), while @CM removed 78.7% at the adsorbent dosage of 1.5 g L^−1^. The results are consistent with the adsorption results previously reported, which showed MoS_2_@CM to have better adsorptive performance. Thus, the above results strongly suggest the practical application of both adsorbents in treating real pharmaceutical wastewaters.

## 4. Conclusions

In this study, a biomass-based adsorbent was successfully produced by doping with MoS_2_ using a hydrothermal process followed by a high-temperature pyrolysis. N_2_ isotherms, SEM-EDS, XRD, and XPS were employed to investigate the effect of the MoS_2_ doping process. MoS_2_@CM exhibited remarkable changes in comparison with the pristine @CM, showing a rougher morphology and irregular surface, indicating a more structural defect. XPS data showed the abundant presence of molybdenum and sulfur states, highlighting its successful doping/modification in comparison with the pristine carbon (@CM). The MoS_2_@CM exhibited a much faster kinetic of adsorption and its maximum adsorption capacity was 424 mg g^−1^ at 45 °C. Besides, the equilibrium studies showed that MoS_2_@CM has higher affinity in adsorbing DCF molecules due to its abundant available adsorption sites to bind DCF. Based on indirect evidence from pH effect, zeta potential, kinetic and isotherm data, as well as literature on MoS_2_ doped carbons, we hypothesize that the following interactions may contribute to the adsorption of DCF onto MoS_2_@CM: pore filling, hydrogen bonding, π–π interactions, and possible electrostatic attractions. Both @CM and MoS_2_@CM can be regenerated using an eluent composed of 0.1 M NaOH + 20% EtOH, with MoS_2_@CM maintaining approximately 81% of DCF removal after five cycles. When tested in the treatment of a more complex synthetic effluent, @CM and MoS_2_@CM removed of 78.7% and 80.2% of the total of compounds of the effluent, respectively, highlighting its potential applicability to treat real effluents.

## Conflicts of interest

The authors declare no conflicts of interest.

## Supplementary Material

RA-016-D6RA03342C-s001

## Data Availability

The data that support the findings of this study are available from the corresponding author upon reasonable request. Supplementary information (SI) is available. See DOI: https://doi.org/10.1039/d6ra03342c.
