# Supplementary material for: Synthesis of novel and sustainable molybdenum disulfide (MoS2)-doped biomass-carbon adsorbent with high-performance sodium diclofenac removal
Source: RSC Adv. 2026 Jul 21;16(38):41951–62. doi: 10.1039/d6ra03342c (PMC13386137; doi:10.1039/d6ra03342c)
Supplement: Supplementary file 1 [file RA-016-D6RA03342C-s001.pdf]

Supplementary Information (SI) for RSC Advances.
This journal is © The Royal Society of Chemistry 2026

Supplementary Material for **Synthesis of novel molybdenum disulfide (MoS_2)-doped biomass-carbon adsorbent with high-performance sodium diclofenac removal**

Wenyuan Duan[1,2*], Yanlin Li[3], Lashari Najeeb ur Rehman[4], Irineu A.S. de Brum[5], Luis O.P. Silva[6*], Guilherme Luiz Dotto[7], Weslei Ambros[5], Eder C. Lima[5], Glaydson Simões dos Reis[4,6*]

[1] *Xi'an Key Laboratory of Advanced Photo-electronics Materials and Energy Conversion Device, Xijing University, Xi'an 710123, China.*

[2] *Monte-Bianco Diamond Applications Co., Ltd., Foshan 528313,China.*

[3] *School of Materials Science and Engineering, Xi'an University of Architecture and Technology, Xi'an 710055, China.*

[4] *Laboratory of Industrial Chemistry and Reaction Engineering, Faculty of Science and Engineering, Åbo Akademi University, 20500 Åbo/Turku, Finland, Finland.*

[5]*Postgraduate Program in Mine, Metallurgical, and Materials Engineering (PPGE3M), School of Engineering, Federal University of Rio Grande do Sul (UFRGS), Av. Bento Gonçalves, 9500 Porto Alegre, RS, Brazil.*

[6]*Universidad de La Costa, CUC, Calle 58 # 55–66, Barranquilla, Atlántico, Colombia.*

[7] *Federal University of Santa Maria, Chemical Engineering Department, Roraima Avenue, Postal Code 1000, 97105–900 Santa Maria, RS, Brazil.*

*Correspondence: lfsoacademico@gmail.com, glaydson.simoesdosreis@abo.fi

S2.Calculation of the adsorption capacity (q) and percentage of removal (%)

The adsorption capacity as a function of time (q_t, mg g^{-1}), adsorption capacity in the equilibrium (q_e, mg g^{-1}), and percentage of removal of sodium diclofenac (DCF) were determined by Equations 1 to 3, respectively:

$$q_t = \frac{(C_0 - C_t)}{m}V \quad \text{(S1)}$$

$$q_e = \frac{(C_0 - C_e)}{m}V \quad \text{(S2)}$$

$$Removal(\%) = 100\left(\frac{C_o - C_e}{C_0}\right) \quad \text{(S3)}$$

Where C_0 is the initial DCF concentration in the liquid phase (mg L^{-1}), C_t is the DCF concentration at time t (mg L^{-1}), C_e is the DCF concentration at equilibrium (mg L^{-1}), m is the amount of adsorbent (g), and V is the volume of DCF solution (L).

S3 kinetics and isotherms models and statistical evaluation

Kinetic models

The kinetic models of pseudo-first-order (PFO, Eq. 4) and pseudo-second-order (PSO, Eq. 5) were used to adjust the experimental data, according to Equations 4 and 5, respectively.

$$q_t = q_1\left(1 - exp(- k_1t)\right) \quad \text{(S4)}$$

$$q_t = \frac{k_2.q_2^2.t}{1 + q_2.k_2.t} \quad \text{(S5)}$$

Where k_1 (min^{-1}), k_2 (g mg^{-1} min^{-1}), and k_{Av} (min^{-1}) are the pseudo-first-order and pseudo-second-order kinetic rate constants, respectively, q_1, q_2, and q_{Av} are the theoretical values for adsorption capacity (mg g^{-1}).

Isotherm models

The adsorption equilibrium data were fitted according to the non-linear Freundlich (Eq. 6) and Langmuir (Eq. 7) isotherm models.

$$q_e = K_F C_e^{1/nF} \quad \text{(S6)}$$

$$q_e = \frac{q_{max} K_L C_e}{1 + (K_L C_e)} \quad \text{(S7)}$$

Where k_F is the Freundlich constant (mg g^{-1})(mg L^{-1})$^{-1/nF}$, $1/n_F$ is the heterogeneity factor, q_m is the maximum adsorption capacity (mg g^{-1}), k_L is the Langmuir constant (L mg^{-1}), q_{max} is the maximum adsorption capacity of the Langmuir model (mg g^{-1}), and C_e is the maximum adsorption capacity at equilibrium.

Statistical evaluation of the fitted models

The parameters of the kinetic and adsorption equilibrium models were determined by non-linear regression through the minimization of the least squares function using the Quasi-Newton method. Calculations will be performed using the Origin software (OriginPro 2016 SR0). The adequacy of the kinetic and equilibrium models was statistically assessed employing the adjusted determination coefficient (R^2_{adj}) and the standard deviation of residues (SD) shown in equations 8 and 9 below.

$$R^2_{adj} = 1 - (1 - R^2).\left(\frac{n-1}{n-p-1}\right) \quad \text{(S8)}$$

$$SD = \sqrt{\left(\frac{1}{n-p}\right).\sum_{i}^{n}(q_{i,exp} - q_{i,model})^2} \quad \text{(S9)}$$

Where $q_{i,\ model}$ is the individual model sorption capacity expected by the model; $q_{i,\ exp}$ is the individual experimentally measured sorption capacity; $\overline{q}_{i,exp}$ is the average of all measured experimental sorption capacities; n is the number of experiments performed; p is the number of model parameters. The R^2_{adj} and SD values were used to compare kinetics and equilibrium models. The best-fitted model would present the R^2_{adj} closer to 1.00 and the lowest SD values.

Regeneration tests

For reusability tests, DCF-laden carbons were washed with water to remove any unadsorbed drug and dried overnight in an oven at 60 °C. Thus, the loaded-DCF carbons were put in contact with .1 M NaOH + 20% EtOH eluent and stirred for 3 hours. The desorbed DCF was then

separated from the @CM and MoS_2@CM adsorbents. Then, they were washed with water to remove the eluent and dried overnight in an oven at 60 °C. The adsorption capacity of both adsorbents were measured repetitively up to five consecutive adsorption–desorption cycles.

Table S1. Composition of the synthetic effluent.

**Drugs**	Concentration in mg L^{-1}
Acetylsalicylic acid	20
Diclofenac	20
Iboprofen	20
Ciprofloxacin	20
Sulfadiazine	20
Metoprocol	20
**Sugars**	
Saccharose	40
Glucose	40
**Other Organic components**	
Urea	20
Humic acid	20
**Inorganic components**	
Ammonium chloride	30
Sodium sulfate	20
Sodium chloride	70
Sodium carbonate	20
Calcium nitrate	20
Potassium nitrate	20
pH	6

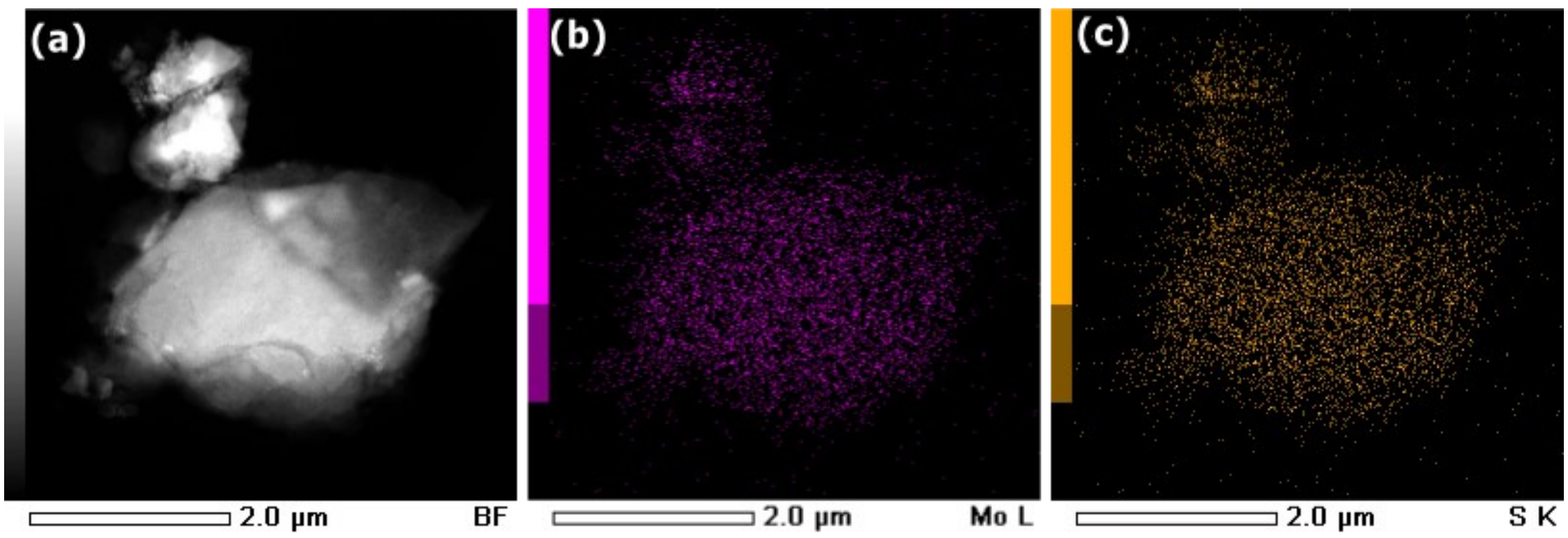

Figure S1 – STEM-EDX mapping of the molybdenum and sulfur elements for MoS_2@CM

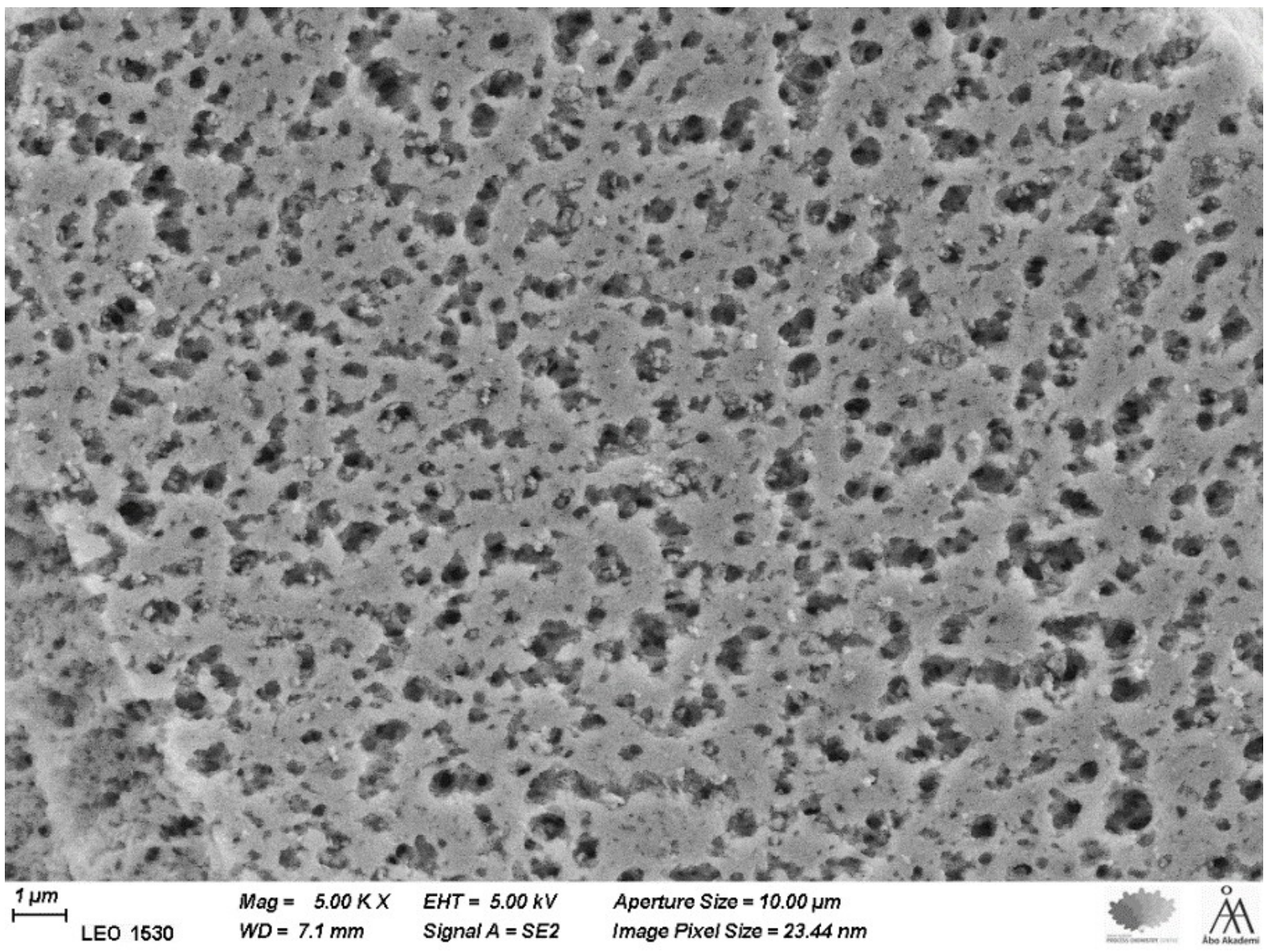

Figure S2 – SEM at high magnification (5.0K) for @CM

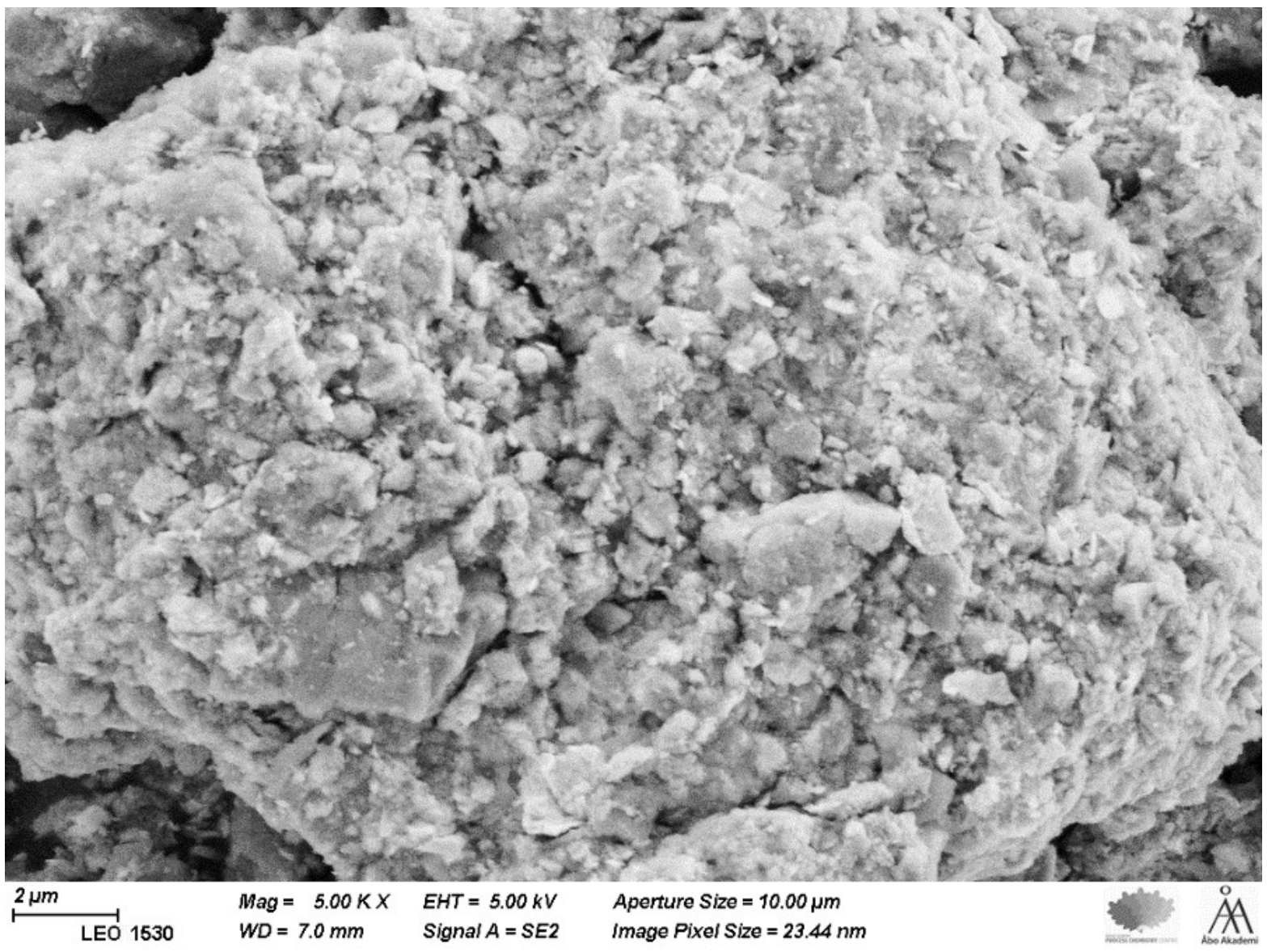

Figure S3 – SEM at high magnification (5.0K) for MoS_2@CM

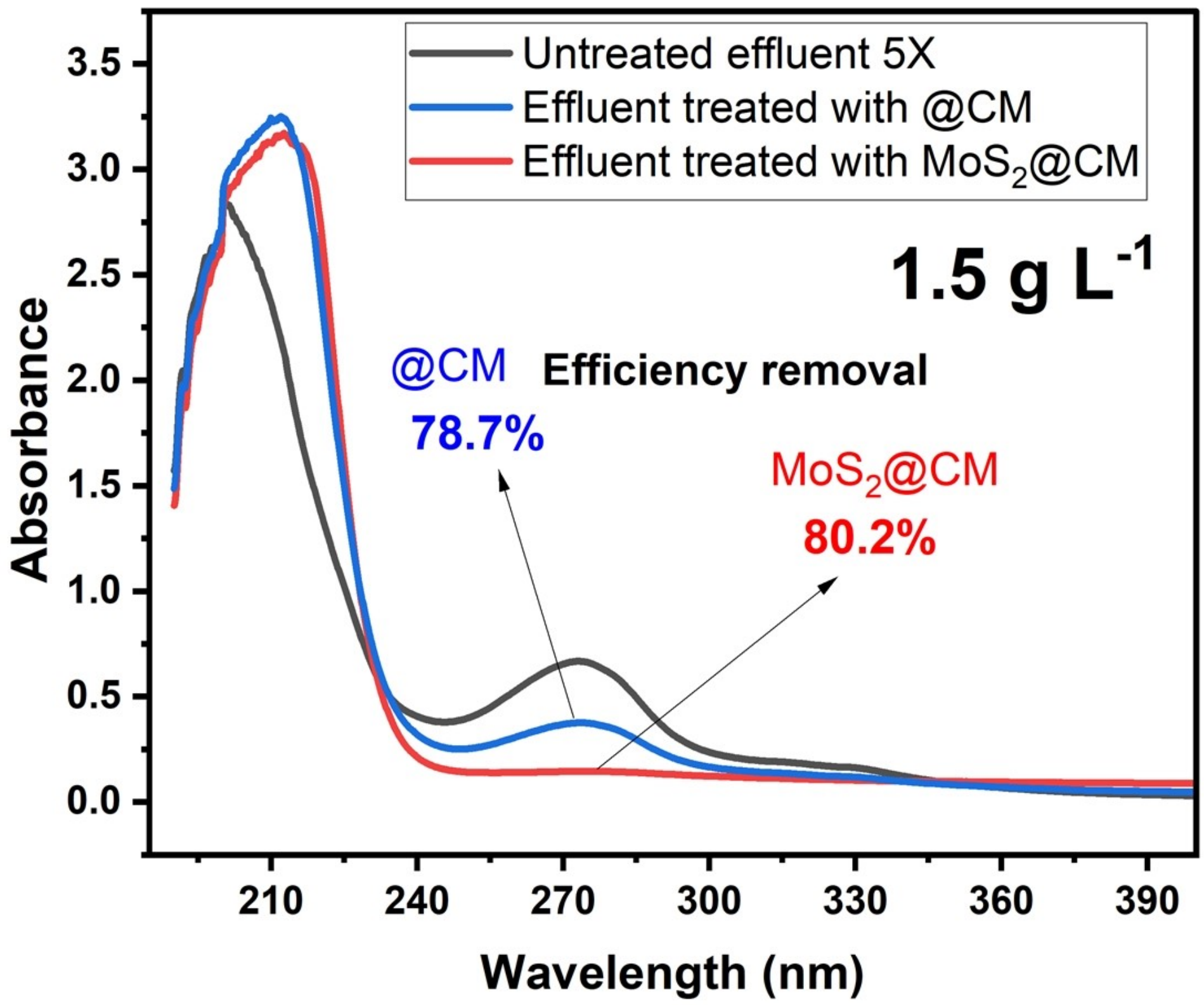

Figure S4. Effluents spectra of @CM and MoS_2@CM materials at a dosage of 1.5 g L^{-1}.
